# A quantitative assessment of aorta root rotation in patients with tetralogy of Fallot evaluated by MSCT

**DOI:** 10.1038/s41598-021-93814-4

**Published:** 2021-07-12

**Authors:** Soha Romeih, Alaa Kaoud, Mohamed Hashem, Mohamed Abdelfattah, Mohamed Gibreel, Mohamed Elzoghby, Mahmoud Shaaban, Wesam El Mozy

**Affiliations:** 1 Department of Radiology, Aswan Heart Centre, Kasser Elhagar Street, P.O Box 81512, Aswân, Egypt; 2 Department of Cardiology, Tanta University, Tanta, Egypt; 3 Department of Anatomy and Embryology, Assiut University, Asyût, Egypt; 4 Department of Radiology, Al-Azhar University, Cairo, Egypt; 5 Department of Radiology, Cairo University, Cairo, Egypt; 6 Department of Anatomy and Embryology, Aswan University, Aswan, Egypt

**Keywords:** Anatomy, Cardiology, Heart development

## Abstract

Lack of conal rotation and conal malseptation is a characteristic anatomical feature for TOF which lead to dextroposed position of aorta and significant RVOT narrowing. The quantitative assessment of these anatomical features using modern cardiac imaging modality has been rarely discussed in the literature. All TOF scanned had in our center from 2013 till 2019 were included. The angle of aortic root rotation was recorded by measuring the angle between a line connecting the midpoint of the non-coronary sinus to the anterior commissure and another line along the interatrial septum. Rotation angles were correlated with proximal main pulmonary artery (MPA) size indexed to BSA. 287 TOF patients were included, 258 patients (91%) had TOF with pulmonary stenosis (TOF-PS) including 138 male (54%), median age 2 years (2 months–40 years), and 29 patients (9%) had TOF with pulmonary atresia (TOF-PA) including 17 male (59%), median age 5 years (1 m-33 years). The whole cases demonstrated clockwise rotation of the aortic root. The mean rotation angle in TOF-PS group was 52.6 ± 20.9° and in TOF-PA group was 64.9 ± 13.9°. Proximal MPA diameter was 11.1 ± 5.9 mm/m^2^. There was a significant negative correlation between aortic root rotation angle and proximal MPA diameter (*r* = − 0.262, *P* = 0.000). The rotation angle of aortic root was significantly higher in TOF-PA compared to TOF-PS (64.9 ± 13.9° vs. 52.6 ± 20.9°, *P* = 0.001, respectively). MSCT provide a quantitative measurement methodology of conal malseptation and its effect in TOF patients. There is a clockwise rotation angle of the aortic root in TOF patients that correlates negatively with proximal MPA size. TOF-PA have a larger rotation angle of aortic root.

## Introduction

Tetralogy of Fallot (TOF) is the most common cyanotic congenital heart disease (CHD) and constitutes 3.5% of all CHD^1^. TOF is characterized by the lack of conal rotation and conal malseptation which is anatomically demonstrated by the anterior deviation of the conal septum^2,3^. Kramer et al. first suggested that “the outflow tract of both ventricles is the region where the conus joins the truncus and that resorption and differential growth are responsible for the reduction of the conoventricular flange, making the conus shift from the extreme right toward the midline of the septum where it comes into alignment with the muscular portion of the interventricular septum”^2^.

The lack of this normal conal rotation brings the aorta to a dextro-posterior position during normal embryogenesis^4^. The aortic root is displaced towards the right –dextroposed—as a consequence of two possible mechanisms^5^: first: rightward translation, possibly secondary to the lack of left migration of the conus. Second: clockwise rotation about an eccentric axis, supposed to be secondary to the lack of conal rotation; or a combination of these two mechanisms. De la Cruz and Da Rocha^6^ suggested that conal malseptation occurs at the expense of the pulmonary artery and it is responsible for the degree of pulmonary (infundibular) stenosis. Therefore, in the fetal lifeblood flow path through the aorta and pulmonary valves is unequal and this flow disturbance in might be the cause of right ventricle outflow tract (RVOT) under-development.

The conal malseptation, as a principal anomaly for TOF, has been extensively studied by morphological studies on postmortem specimens^1–6^. However, the objective quantification assessment of the conal malseptation by cardiac imaging modalities is still underutilized. There is one publication in the late 80 s using 2D echocardiography that studied the aortic root displacement to the right (dextroposition) in TOF patients and they measured the values of the aortic valve rotation angles in the normal population compared with TOF patients. The aortic root rotation angle was 23.4 ± 8.3° in normal subjects, while in TOF patients have higher clockwise rotation angles; 59.2 ± 10.7°^2^. The RVOT underdevelopment was not assessed or quantified in this study.

Currently, the utility of multi-slice computed tomography (MSCT) in the structural assessment of the heart has its principle strength in the evaluation of cardiac anatomy. The modern generation of MSCT allows fast imaging with very short acquisition times (often within a single breath-hold) that translates into superior spatial and temporal resolution from diminished motion artifact^7,8^.

We hypothesize that a quantitative assessment of the conal malseptation in TOF patients could be accurately performed using ECG-gated MSCT angiography images. The clockwise angle rotation of the aortic root could be a potential indicator for the severity of conal malseptation and therefore the RVOT maldevelopment.

## Patients and methods

### Study population and data collection

Our study is a retrospective one, cross-sectional, single-center analysis of all TOF patients who underwent MSCT examination, at our center from 2013 to 2019, for clinical purpose. This study was carried out following relevant guidelines. The included TOF patients were either (1) TOF with pulmonary valve stenosis (TOF-PS), who have a forward flow from the RV through a stenotic and narrowed pulmonary valve to the pulmonary artery, (2) TOF with pulmonary atresia (TOF-PA), who have no forward flow from the RV to pulmonary arteries, the pulmonary blood supply depends mainly on patent ductus arteriosus or collaterals. There were 318 patients, all scanned at 128 dual sources multislice SOMATOM scanner (Siemens, Erlangen, Germany). The examination was performed with retrospective ECG gating and tube modulation to reduce the radiation dose. The study was approved by the internal board of Aswan Heart Centre- Magdi Yacoub foundation. Written informed consent was obtained from all included patients to perform the MSCT and if patients are younger than 18 years the informed consent was waived by the Aswan heart centre- Magdi Yacoub foundation medical ethics committee.

### CT imaging protocol

#### Scan protocol and image reconstruction

MSCT scan data were performed from the thoracic inlet to diaphragm in a craniocaudal direction. The parameters of scan include detector collimation of 128–0.6 mm, section collimation of 128–0.6 mm using a z-flying focal spot, gantry rotation time was 0.28 s, the pitch was 0.3–0.4 and it was adjusted according to heart rate and tube potential of 100–120 kVp and temporal resolution is good (75 ms).

Tube current modulation (ECG pulsing on) was applied. During diastole phases (mostly 70–80% of R–R interval) a nominal tube current was used, while during systole (mostly 40–50% of R–R interval) a reduced tube current was used. The use tube current modulation technique leads to a considerable reduction in radiation dose (60% dose reduction). Analysis performed on best systole phase 40% (systole) and best diastole phase 70% (diastole) phases.

### Image analysis and measurements

**Figure 1:**
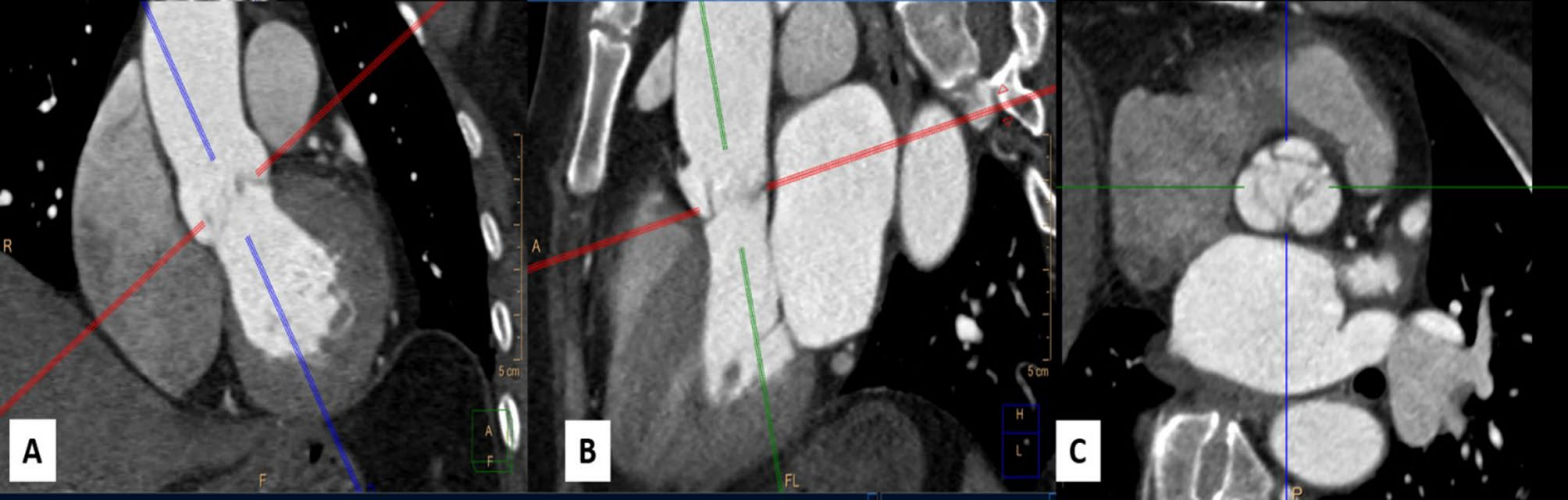
Short axis of aortic valve determination, on best systole phase by perpendicular lines on two orthogonal views of aorta (**A**) coronal view of aorta (**B**) sagittal view of aorta (**C**) short axis of aortic valve.

**Figure 2:**
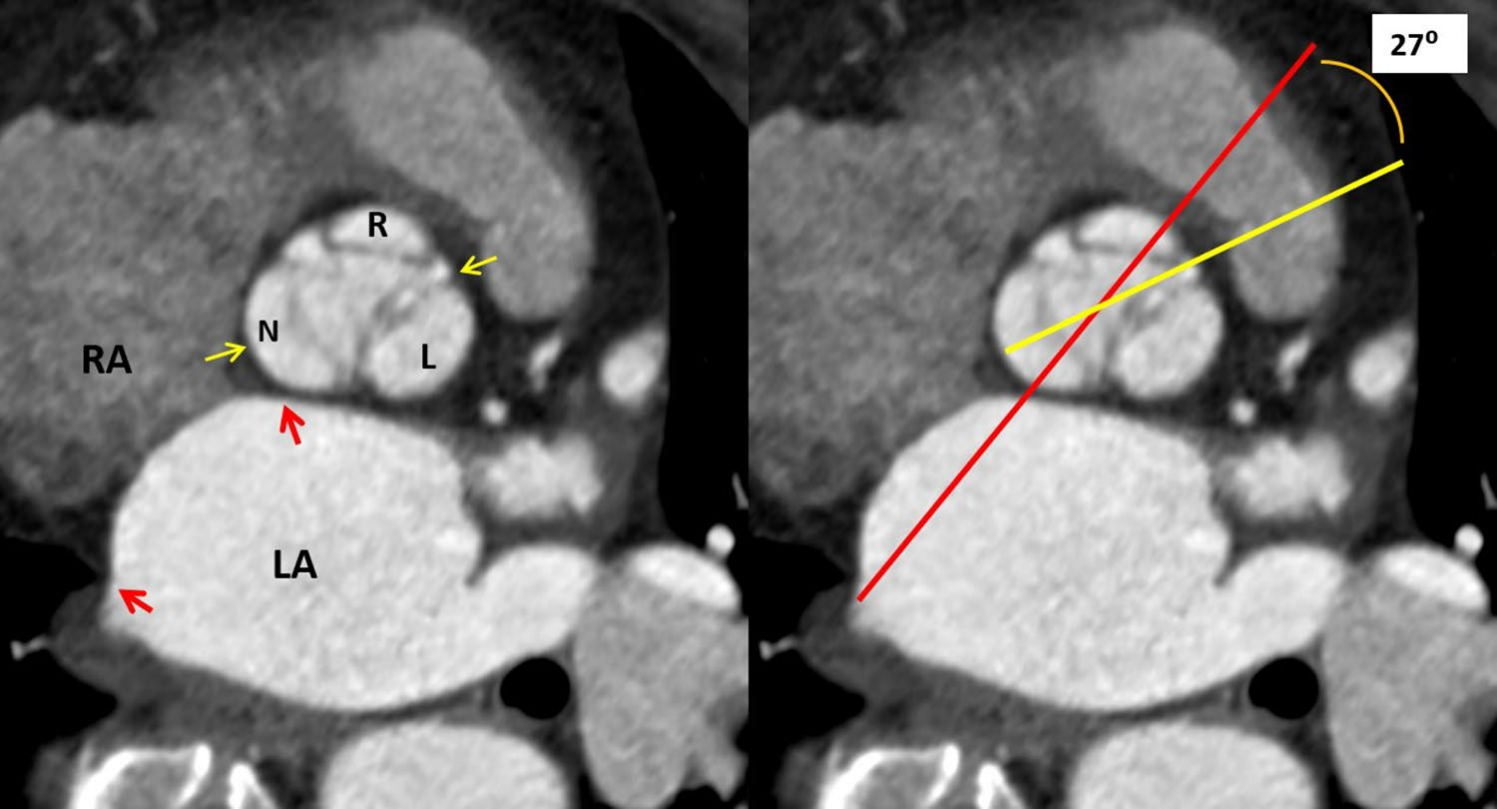
Method of aortic root rotation angle measurement between the red line (the plane of the interatrial septum) and the yellow line (connecting the middle of the non-coronary sinus and the anterior commissure) using true short-axis image of the aortic valve.

Images were reviewed on (Singovia; Medical Systems, Siemens, Erlangen, Germany). The short axis of the aortic root was determined on the best systolic phase by perpendicular lines on two orthogonal planes of the aorta, Fig. 1. The aortic root rotation (in plane) was defined as any clockwise rotation of the aortic root about the centerline of the aortic root. To measure the angle of the aortic root rotation, using a true short-axis view of the aortic valve a line connecting the mid-point of the non-coronary sinus to the interleaflet anterior commissure between the coronary sinuses, as defined in a previous publication^4^, was drawn and its angle with the plane of the interatrial septum (IAS) was measured. The plane of IAS was drawn between the anterior and posterior insertion points of IAS in the left atrium, Fig. 2.

**Figure 3:**
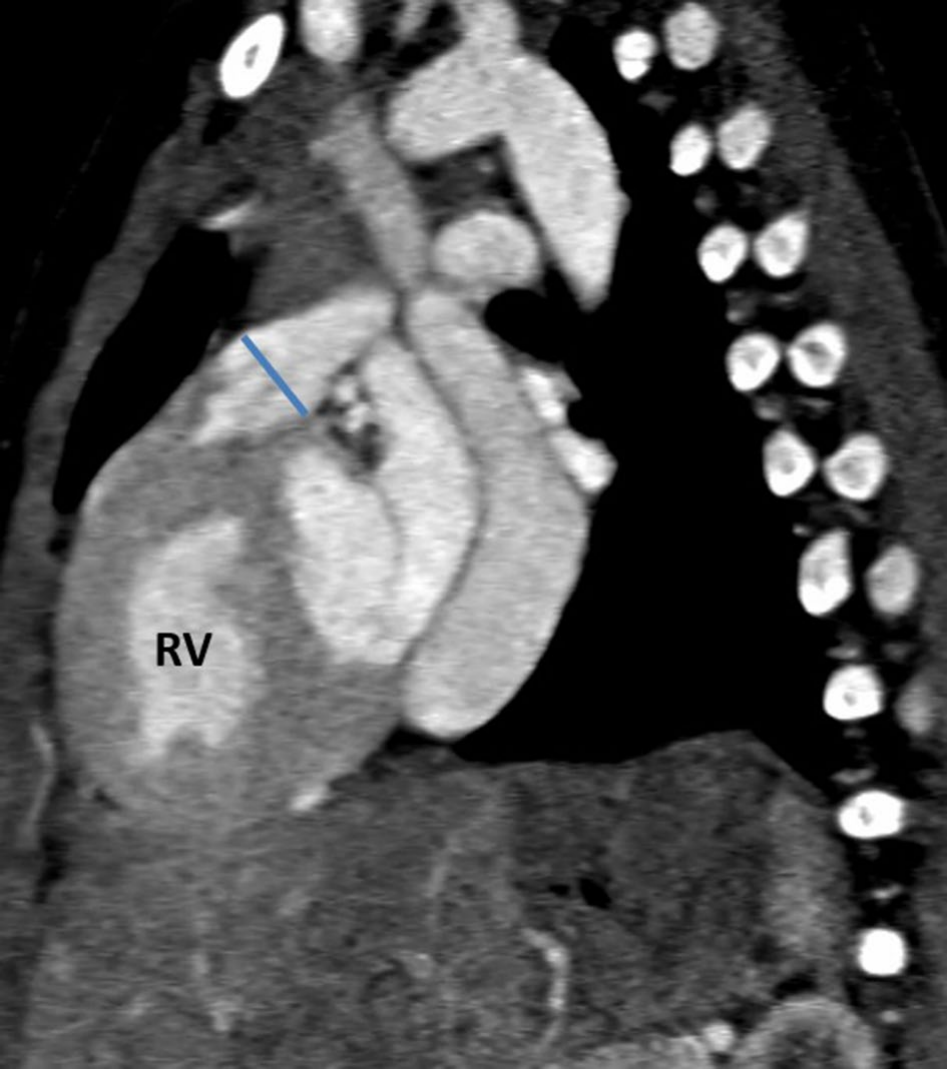
Measurement of the proximal main pulmonary artery (MPA) diameter at the widest diameter before bifurcation, the diameter of MPA is indexed to the BSA.

Proximal MPA diameter was measured on the sagittal plane, the diameter was taken at the widest part before MPA bifurcation during the systole phase. Proximal MPA diameter was indexed for body surface area, Fig. 3.

Measurement reproducibility determined by 2 independent readers in a random sample of 100 subjects were excellent for interobserver and intraobserver variability (interobserver intraclass correlation coefficient: rotation angle of aortic root = 0.90, proximal MPA size = 0.98, and; intraobserver intraclass correlation coefficient: rotation angle of aortic root = 0.97, proximal MPA size = 0.99, respectively).

### Statistical analysis

As the sample size in our study was relatively large, data distribution was checked using histogram. To test the correlation depending on the data distribution, we used Pearson or Spearman correlation.

The relation between aortic angle and size of the MPA was assessed using analysis of variance method (ANOVA). Receiver-Operator-Characteristics (ROC) curve analysis was performed to determine the cut-off value of the aortic angle, above which the patient will be at risk of pulmonary atresia and the area under the curve (AUC) was determined. We used the independent t-test or Wilcoxon rank-sum test for continuous measurement between outcome categories. While, we conducted both parametric and non-parametric statistics for measurements with a borderline normal distribution (i.e. skewed with long tail). Alpha level of 0.05 was used. The Intra-class correlation (ICC) with the absolute agreement was used to inspect the agreement between raters for the approximal MPA measurement rotation and aortic root angle of 100 randomly selected patients.

## Results

### Demographic data

Demographic variables of the study cohort are summarized in Table 1. There were 318 TOF patients. Poor quality images due to severe artifacts (N = 4), poor opacification of the aorta (N = 3) that interfered with measurements, patients who underwent surgical repair of the TOF (N = 16), and patients with absent pulmonary valve(N = 8) were excluded. The total number of the final study cohort was 287 TOF patients, 258 patients (91%) has TOF with pulmonary stenosis (TOF-PS) (group A) and 29 patients (9%) have TOF with pulmonary atresia (TOF-PA) (group B).

**Table 1:** Demographic data of the study cohort.

	TOF patients		Controls
	Group A1 TOF-PS	Group A2 TOF-PA	Group B
Total number	258	29	40
Age (years)	2 years (2 months–40 years)	5 years (1 month–33 years)	25 ± 2 years
Male, number (%)	138 (54%)	17 (59%)	18 (45%)
Female, number (%)	120 (46%)	12(41%)	22 (55%)
BSA (m^2^)	0.68 ± 0.40	0.80 ± 0.48	1.3 ± 0.5
NYHA class	I	I	–
O2 saturation	72 ± 5%	71 ± 7%	100%
Heart rate (beat/min)	100 ± 22	102 ± 19	100 ± 23
MSv in MSCT	0.6 ± 0.5	0.5 ± 0.6	0.6 ± 0.5
Contrast does ml/kg	1 ml/kg	1 ml/kg	1 ml/kg

**Figure 4:**
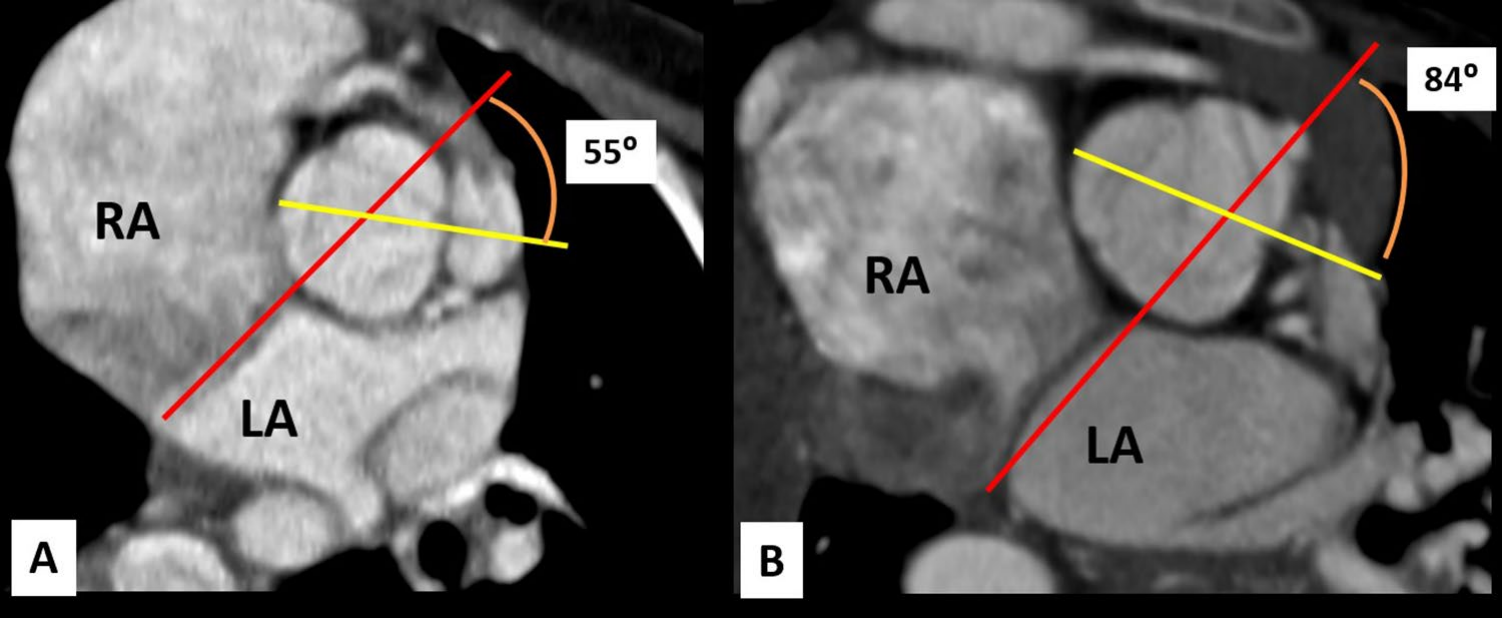
Measurement of aortic root rotation angle in TOF patients. (**A**) A case of TOF-PS with a clockwise rotation angle of the aortic root is 55°. (**B**) A case of TOF-PA with the extreme clockwise rotation angle of the aortic root is 84°.

**Figure 5:**
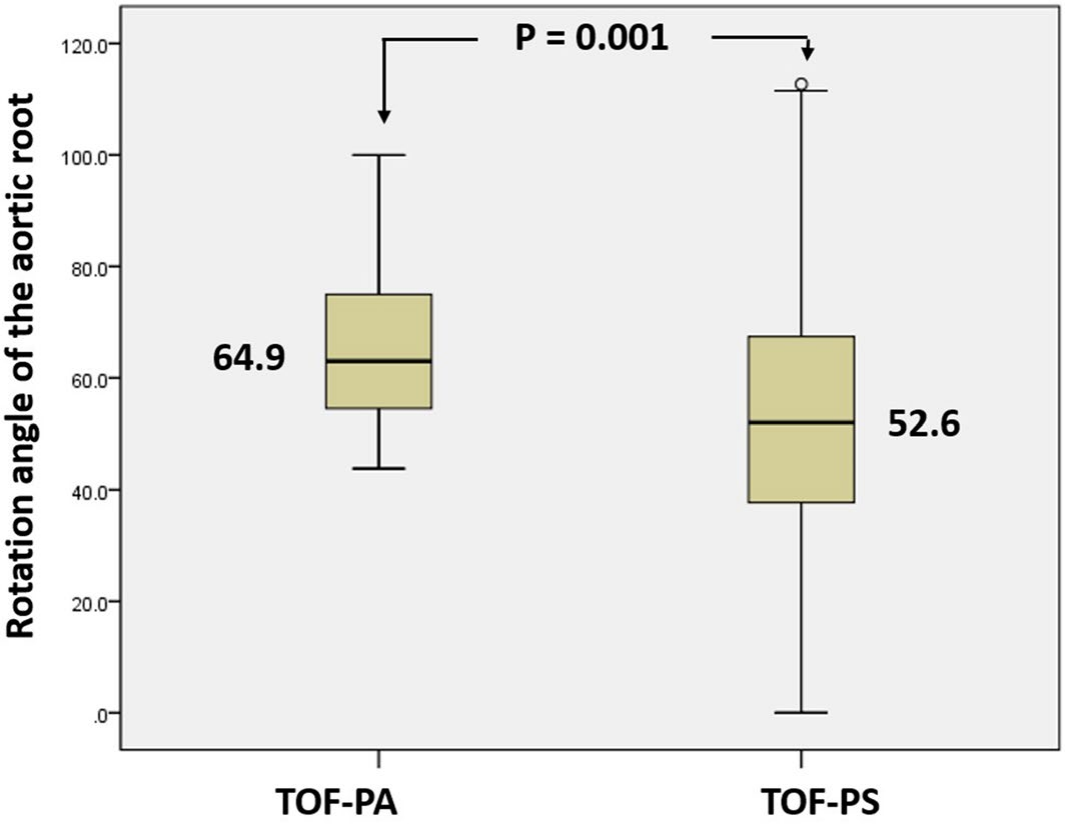
Plot-Box showed the mean of the clockwise rotation angle of aorta root of TOF-PA and TOF-PS which showed a significant difference.

The whole TOF patients demonstrated a clockwise rotation of the aortic root with no cases with counterclockwise rotation, Fig. 4. The mean rotation angle of the aortic root was 52.6 ± 20.9° in the TOF-PS (Group A) and 64.9 ± 13.9° in TOF-PA (Group B). The rotation angle of the aortic root was significantly higher in TOF-PA compared to TOF-PS (64.9 ± 13.9 vs. 52.6 ± 20.9, *P* = 0.001 respectively), Fig. 5.

**Figure 6:**
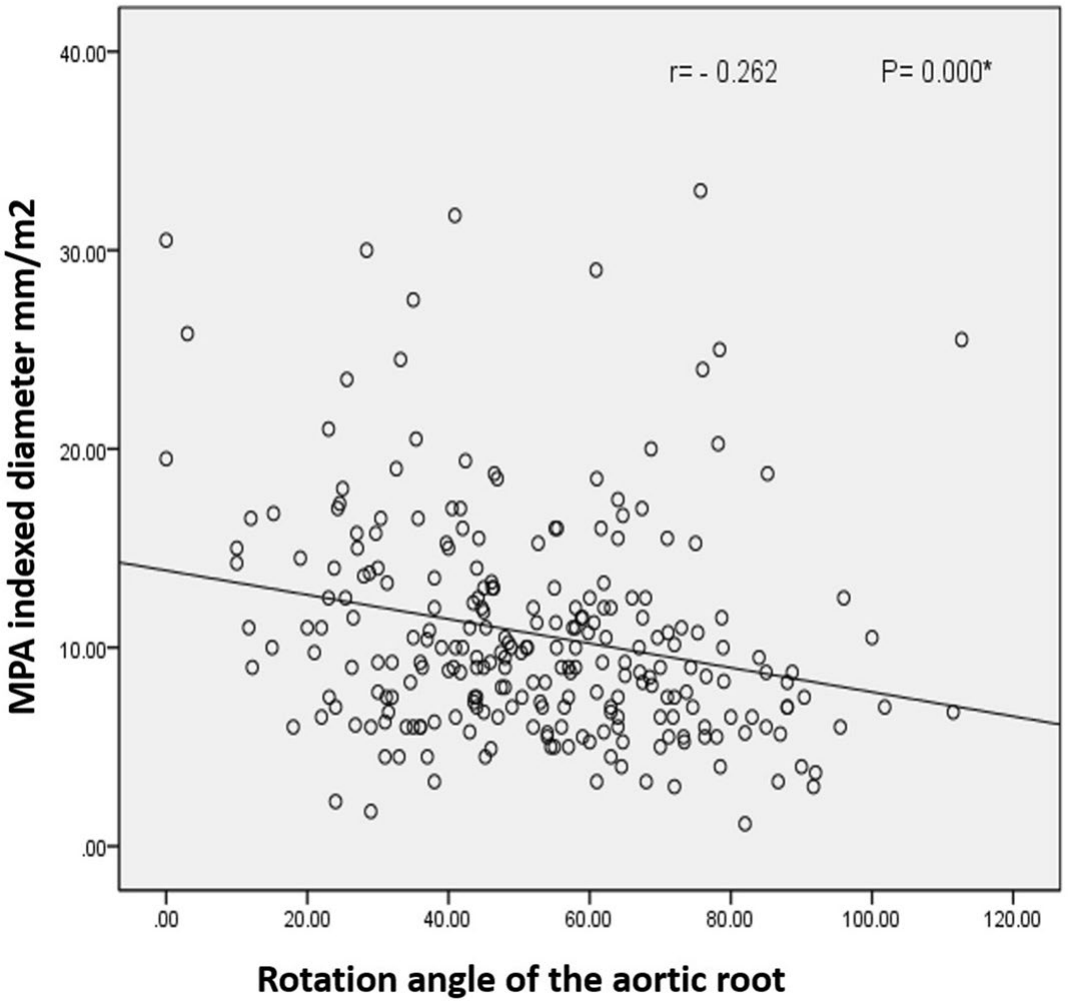
A negative correlation between the clockwise angle rotation of aortic root and MPA indexed diameter.

In TOF-PS (Group A1), MPA diameter indexed to BSA was 11.1 ± 5.9 mm/m2. There was a negative correlation between the clockwise rotation angle of the aortic root and the indexed MPA diameter (*r* =− 0.262, *p* = 0.000, regression analysis = 0.028), Fig. 6.

**Figure 7:**
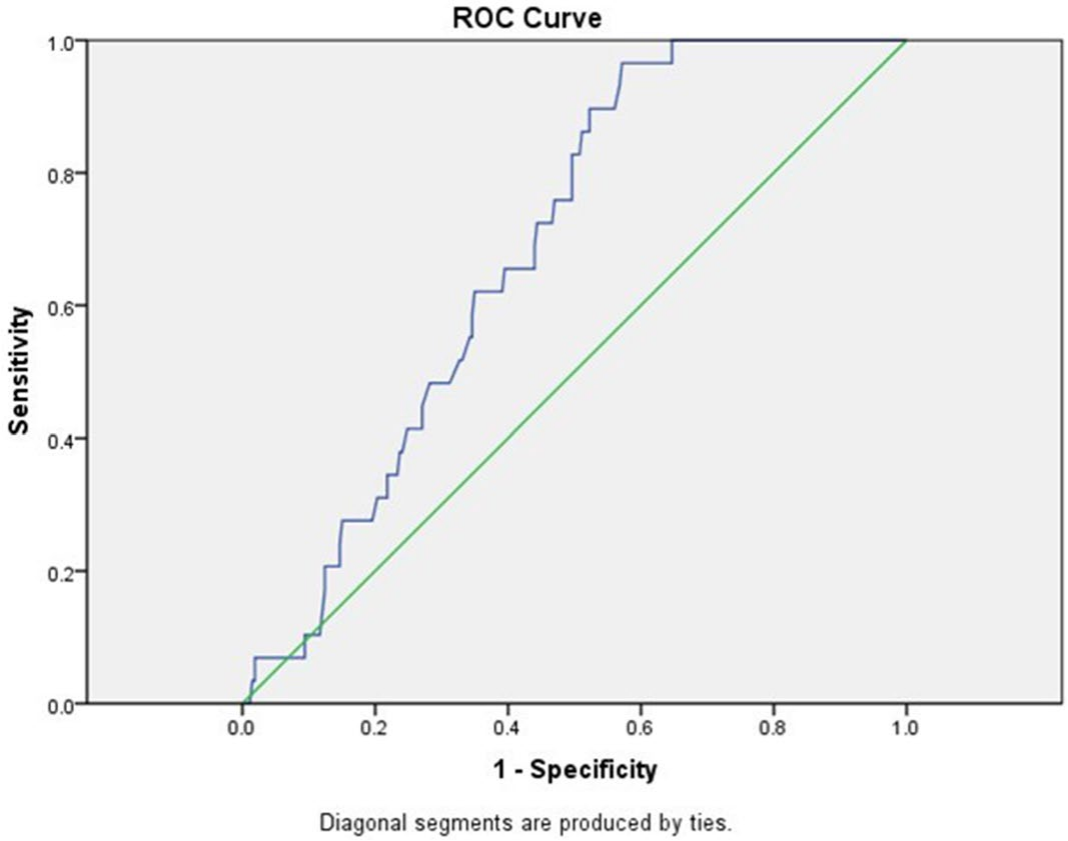
Receiver operator characteristic curve (ROC) of the relation between aortic angle and TOF-PA. A cut-off value of aortic angle ≥ 58.4° yielded a sensitivity of 66% and specificity of 61%.

A cut-off value of aortic angle of 58.4° was determined, above which there is a risk of pulmonary atresia (AUC = 0.685, 95% CI = 0.61–0.76) with sensitivity 66%, specificity 61%, positive predictive value was 66% and negative predictive value was 60%, Fig. 7.

## Discussion

Our data confirmed the clockwise rotation of the aortic root in TOF patients due to conal malseptation. We also demonstrated a negative correlation between the clockwise rotation angle of the aortic root with the proximal MAP indexed diameter. TOF-PA has a higher rotation angle of aortic root than TOF with pulmonary stenosis, our study proposed a cut-off rotation angle of the aortic root (58.4°), with moderate sensitivity and specificity, above which the TOF patients are likely to have atretic pulmonary valve instead of stenotic pulmonary valve.

Our results are in agreement with findings in the morphologic studies conducted by Becker et al.^9^ and Goor and associates, who performed virtually identical geometric measurements on postmortem specimens^10–12^. The aorta dextroposition which indicates a pathologic condition with a rightward displacement of the aortic root and the clockwise rotation angle of the aortic root confirms the dextroposition of the aorta. Previously, the terms ‘‘dextroposition’’ and ‘‘overriding’’ have been used interchangeably. However, overriding of the aorta over the RV; which occurs in the normal heart; should not be confused with dextroposition. The existence and extent of dextroposition of the aorta in the tetralogy of Fallot are important as it affects the further treatment strategy.

Our data is also in agreement with the 2D echocardiography study performed on 22 TOF patients, which demonstrated a clockwise rotation with a rightward displacement of the aortic root due to the lack of conal rotation^2^. However, no assessment of RVOT development was performed in this study which could be explained by the limited acoustic window of echocardiographic examination.

To our best knowledge, our study is the first study to document that the clockwise rotation angle of the aortic root, due to conal malseptation, is associated with a smaller MPA indexed diameter. These findings support the morphological studies that extensively discussed the geometric features of conal malseptation in TOF^13–16^. Studies in chick embryos by Steding and Seidl^13^ were able to demonstrate that abnormal angulation of the conotruncal border region, created experimentally by a microfilament sling, caused the flow path to be divided unequally, and that flow disturbance may be responsible for TOF malformation.

Furthermore, data by Icardo and Manasek u^15,16^ suggested that the alterations inflow may influence neural crest cell migration and/or cellular production of fibronectin, an extracellular matrix glycoprotein, which plays a key role in morphogenesis of cardiac and other tissues. This may be responsible for subsequent aberrations in conus underdevelopment. This could explain our results that TOF with pulmonary atresia has a significantly higher rotation angle of the aortic root compared to TOF without pulmonary atresia.

Therefore, we believe that our data provide an objective quantitative measurement methodology using the modern cardiac technique for what was previously stated in the morphological studies of TOF disease.

## Study limitations

1. Sample size of TOF-PA is small because many of those patients presented in critical clinical status. Therefore, many will have cardiac catheterization rather than MSCT.
2. The proposed cut-off rotation angle of aortic root (58.4°) above which the TOF patients will have atretic pulmonary rather than stenotic pulmonary valve had moderate sensitivity and specificity due to lower sample size. Further multicenter studies could help in the validation of this point more
3. Correlation between the proposed cut-off rotation angle of aortic root and the prognosis of TOF patients is a point for further multi-centers research. We were not able to perform this correlation because many of the TOF patients presented in our territory center from the counter and many African countries for imaging purposes, therefore, their follow-ups were lost.

## Conclusion

MSCT provides a quantitative measurement methodology of conal malseptation and its effect in TOF patients. There is a clockwise rotation angle of the aortic root in TOF patients. The clockwise rotation angle of the aortic root correlates negatively with the size of the proximal main pulmonary artery. TOF patients and pulmonary atresia have a larger rotation angle of the aortic root.
